# Archaeal Chromatin Proteins Cren7 and Sul7d Compact DNA by Bending and Bridging

**DOI:** 10.1128/mBio.00804-20

**Published:** 2020-06-09

**Authors:** Zhenfeng Zhang, Zhengyan Zhan, Bing Wang, Yuanyuan Chen, Xiuqiang Chen, Cuihong Wan, Yu Fu, Li Huang

**Affiliations:** a State Key Laboratory of Microbial Resources, Institute of Microbiology, Chinese Academy of Sciences, Beijing, China; b Hubei Key Lab of Genetic Regulation & Integrative Biology, School of Life Sciences, Central China Normal University, Wuhan, China; c National Key Laboratory of Biomacromolecules, Institute of Biophysics, Chinese Academy of Sciences, Beijing, China; d College of Life Science, University of Chinese Academy of Sciences, Beijing, China; University of Vienna

**Keywords:** *Archaea*, atomic force microscopy, chromatin protein, single-molecule technology, *Sulfolobus*

## Abstract

Archaeal chromatin proteins Cren7 and Sul7d from *Sulfolobus* are DNA benders. To better understand their architectural roles in chromosomal DNA organization, we analyzed DNA compaction by Cren7 and Sis7d, a Sul7d family member, from Sulfolobus islandicus at the single-molecule (SM) level by total single-molecule internal reflection fluorescence microscopy (SM-TIRFM) and atomic force microscopy (AFM). We show that both Cren7 and Sis7d were able to compact singly tethered λ DNA into a highly condensed structure in a three-step process and that Cren7 was over an order of magnitude more efficient than Sis7d in DNA compaction. The two proteins were similar in DNA bending kinetics but different in DNA condensation patterns. At saturating concentrations, Sis7d formed randomly distributed clusters whereas Cren7 generated a single and highly condensed core on plasmid DNA. This observation is consistent with the greater ability of Cren7 than of Sis7d to bridge DNA. Our results offer significant insights into the mechanism and kinetics of chromosomal DNA organization in Crenarchaea.

## Abstract (precis)

A long-standing question is how chromosomal DNA is packaged in Crenarchaeota, a major group of archaea, which synthesize large amounts of unique small DNA-binding proteins but in general contain no archaeal histones. In the present work, we tested our hypothesis that the two well-studied crenarchaeal chromatin proteins Cren7 and Sul7d compact DNA by both DNA bending and bridging. We show that the two proteins are capable of compacting DNA, albeit with different efficiencies and in different manners, at the single molecule level. We demonstrate for the first time that the two proteins, which have long been regarded as DNA binders and benders, are able to mediate DNA bridging, and this previously unknown property of the proteins allows DNA to be packaged into highly condensed structures. Therefore, our results provide significant insights into the mechanism and kinetics of chromosomal DNA organization in Crenarchaeota.

## INTRODUCTION

Chromosomal DNA organization is critical to all living organisms since the extraordinarily long genomic DNA must be packaged into the small volume of a nucleus or a cell while remaining accessible to the molecular machineries functioning in DNA transactions. Chromatin proteins are responsible for this task and are known to fulfill it mainly by DNA wrapping, bending, or bridging or by forming protein-DNA filaments (1). *Archaea* have evolved a variety of chromatin proteins with distinct phylogenetic distributions. Archaeal histones, homologues of the eukaryotic histone H3/H4, are present in a number of archaeal phyla. These proteins compact DNA in a manner similar to that seen with eukaryotic histones, wrapping DNA around the core tetramer/hexamer to form nucleosome-like or filamentous structures (2, 3). Intriguingly, archaeal histones are absent from all crenarchaeal genera except for *Thermofilum*, *Vulcanisaeta*, and *Caldivirga* (4). Instead, several small, abundant, and basic DNA-binding proteins have been isolated from crenarchaea. Among them, Cren7 is the most highly conserved (5). The only known crenarchaeal species that lacks a Cren7 homologue is Thermofilum pendens Hrk5, which encodes an archaeal histone (6). On the other hand, both *Vulcanisaeta* and *Caldivirga* contain not only an archaeal histone but also a Cren7 homologue (4). Therefore, crenarchaea may employ a Cren7-based strategy in chromosomal DNA organization. Additional chromatin proteins with narrower phylogenetic distributions have also been identified (7–9). For example, members of the Sul7d protein family, existing only in *Sulfolobales*, are among the most extensively studied chromatin proteins from *Archaea* (4). Considerable efforts have been devoted to the understanding of the functional roles of these chromatin proteins in genome organization in crenarchaea. Proteins of the Sac10b family, also known as Alba, are highly conserved in *Archaea*. However, members of this protein family from *Sulfolobus* appear to be able to bind RNA *in vivo* (10, 11), although they were shown previously to compact DNA *in vitro* (12). Therefore, the functional role of the Sac10b proteins remains to be established.

It has been shown that Cren7 and Sso7d, a Sul7d family protein, are synthesized in abundance (∼1% and ∼5% of total cellular proteins, respectively) in the hyperthermophilic crenarchaeon Sulfolobus solfataricus (5, 13). The two proteins are in general similar in biochemical property and tertiary structure. Both proteins preferentially bind double-stranded DNA (dsDNA) over single-stranded DNA (ssDNA), protect duplex DNA from thermal denaturation, and constrain negative DNA supercoils *in vitro* (5, 14). Sso7d was also found to stimulate the intramolecular ligation of a 129-bp fragment, indicating the ability of the protein to induce DNA bending in solution (14). Structural studies showed that both proteins adopt an SH3-like fold, binding DNA in the minor groove and inducing a single-step sharp kink into DNA (approximately 50° to 60°) (15–19). A single-molecule (SM) analysis performed using magnetic tweezers revealed that Cren7 and Sso7d compact DNA to similar extents by introducing rigid bends (20). These results are consistent with the notion that the two proteins are DNA benders. However, the proteins appear to introduce more conformational changes than bending in DNA as suggested by their ability to constrain DNA in negative supercoils (5). The roles of the two proteins in DNA organization were further complicated by the recent finding that Cren7 and Sso7d induced opposite changes in the writhe of the axis of the bound DNA, presumably forming protein-DNA filaments in different patterns (21). Furthermore, Sso7d bound more strongly to GC-rich DNA sequences than to AT-rich sequences (22, 23), whereas Cren7 showed the opposite binding preference (21). Therefore, it is of interest to determine if and how Cren7 and Sul7d serve distinct roles in chromosomal organization.

In the present report, we investigated the architectural roles of Cren7 and Sis7d, a Sul7d family protein from S. islandicus, in chromosomal organization using single-molecule total internal reflection fluorescence microscopy (SM-TIRFM) and atomic force microscopy (AFM). We show that both Cren7 and Sis7d were able to compact DNA into a highly condensed structure and that the former was significantly more efficient in DNA compaction than the latter. We demonstrate for the first time that the two proteins were capable of DNA bridging and that both DNA binding and bridging were involved in DNA compaction. A model is proposed for DNA packaging by Cren7 and Sul7d in crenarchaea.

## RESULTS

### Efficient DNA compaction by Cren7 and Sis7d.

**FIG 1:**
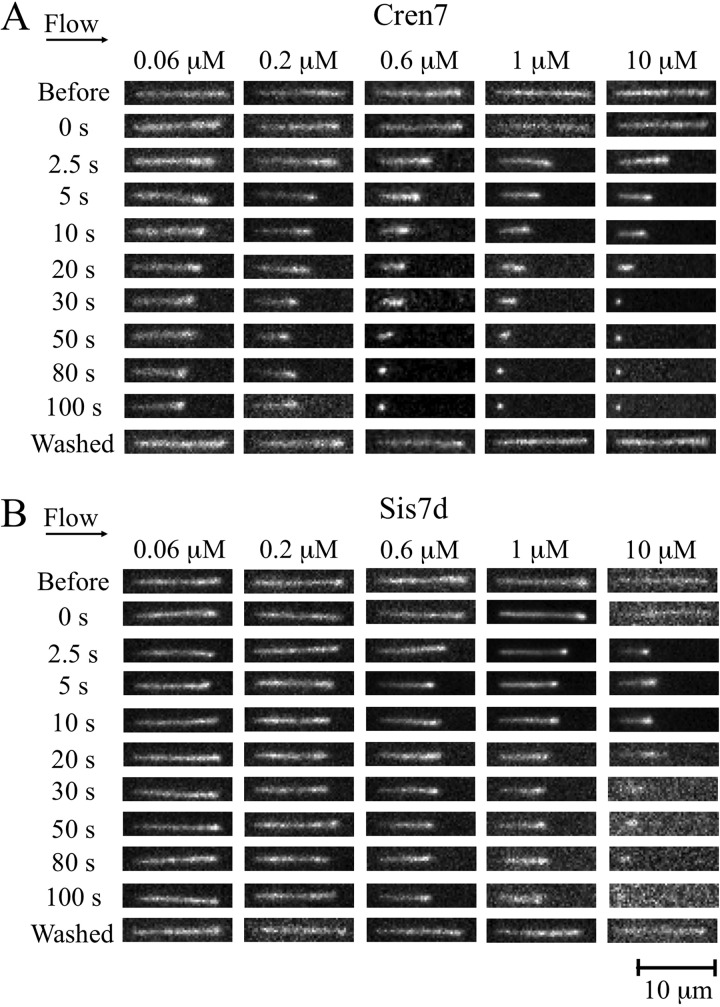
SM-TIRF visualization of the DNA compactions induced by Cren7 (A) and Sis7d (B) at different protein concentrations. Frames representing the indicated time points of a representative single λ DNA molecule from the video recorded at each protein concentration are shown plotted as a montage.

To visualize the influence of binding by Cren7 or Sis7d on the conformation of DNA at the single-molecule level by total internal reflection fluorescence microscopy (TIRFM), linear λ DNA molecules were tethered at one end to a polyethylene glycol (PEG)-passivated microfluidic flow cell via a biotin-streptavidin (SA) interaction, stretched by the use of a constant flow of buffer, and labeled with Sytox orange fluorescent dye (10 nM). In our preliminary experiments, we found that λ DNA was well extended at a flow rate of 100 μl/min (Fig. 1, top panels). In our assays, which involved the use of flow-stretched DNA molecules, the tension of the DNA was highest at the point of tethering (estimated to be >1 pN at the flow rate of 100 μl/min) and decreased to zero at the free end. The mean end-to-end length of the naked DNA was determined to be 13.59 ± 0.16 or 13.27 ± 0.13 μm (*n* = 300) in Sis7d or Cren7 assays, respectively (see Fig. S1 in the supplemental material), or ∼80% of the theoretical length of the DNA (∼16.49 μm). Injection of either Cren7 or Sis7d was accompanied by a reduction in the contour length of the DNA (Fig. 1; see also Movies S1 to S10 at http://nmdc.cn/resource/attachment/detail/NMDCX0000001). The size change of the DNA induced by the binding of Cren7 or Sis7d did not result from the breakage or from other unknown covalent changes of the DNA since the mean length of the DNA remained largely unaltered (13.82 ± 0.11 and 13.61 ± 0.17 μm for the assays performed with Sis7d and Cren7, respectively; *n* = 300) after the removal of the protein with 0.1% SDS (Fig. 1, bottom panels) or 0.5 to 1 M KCl (data not shown). Therefore, we conclude that both Cren7 and Sis7d were able to compact DNA in a reversible manner.

10.1128/mBio.00804-20.2 FIG S1 Gaussian fits of the end-to-end length of λ DNA molecules (*n* = 300) before injecting of protein (dashed lines) and after washing the bound proteins with 0.1% SDS (solid lines) for assays with Sis7d (A) or Cren7 (B). Download FIG S1, JPG file, 0.4 MB. Copyright © 2020 Zhang et al. 2020 Zhang et al. This content is distributed under the terms of the Creative Commons Attribution 4.0 International license.

To compare the abilities of Cren7 and Sis7d to compact DNA, we then carried out the DNA compaction assays on the two proteins at different protein concentrations (0.06, 0.2, 0.6, 1.0, and 10 μM). Snapshots of individual protein-bound DNA molecules taken at the indicated time points in each experiment are shown in Fig. 1. As the concentration of either protein increased, the minimum length of the DNA decreased. While the contour length of the DNA was reduced by 30% at 0.06 μM Cren7, it was shortened by ∼50% at 0.2 μM Cren7 within 100 s of the injection of the protein. The DNA molecules were nearly completely compacted into highly condensed globular structures (<2 μm in dia.) in 80 s in the presence of 0.6 μM Cren7 (Fig. 1A). At still higher Cren7 concentrations, the DNA was shortened faster and condensed into a tighter structure. For instance, DNA molecules were condensed by Cren7 at 10 μM into globular structures within 30 s (Fig. 1A). By comparison, Sis7d was less efficient than Cren7 in DNA compaction (Fig. 1B). Injection of 0.2, 0.6, and 1.0 μM Sis7d led to the shortening of the DNA by ∼10%, 30%, and 40%, respectively. Highly condensed structures were formed only at 80 s following the injection of 10 μM Sis7d (Fig. 1B). The DNA compaction assays were also performed at a flow rate of 50 μl/min (Fig. S2). Within 100 s of the protein injection, the contour length of the DNA was reduced by ∼60% at 0.2 μM Cren7 and by ∼50% at 1.0 μM Sis7d, suggesting that the DNA was more readily compacted by the two proteins with lower stretch tension along the singly tethered DNA molecule.

10.1128/mBio.00804-20.3 FIG S2 Effect of the flow rate on DNA compaction by Cren7 (0.2 μM) and Sis7d (1.0 μM) as visualized by SM-TIRF. Frames representing the indicated time points of a representative single λ DNA molecule from the video recorded are shown as a montage. The normalized final length of DNA after compaction (*L*_f_), representing an average of the measurements of 20 DNA molecules for each experiment, is also shown. Download FIG S2, JPG file, 0.6 MB. Copyright © 2020 Zhang et al. 2020 Zhang et al. This content is distributed under the terms of the Creative Commons Attribution 4.0 International license.

### Kinetics of DNA compaction by Cren7 and Sis7d.

**FIG 2:**
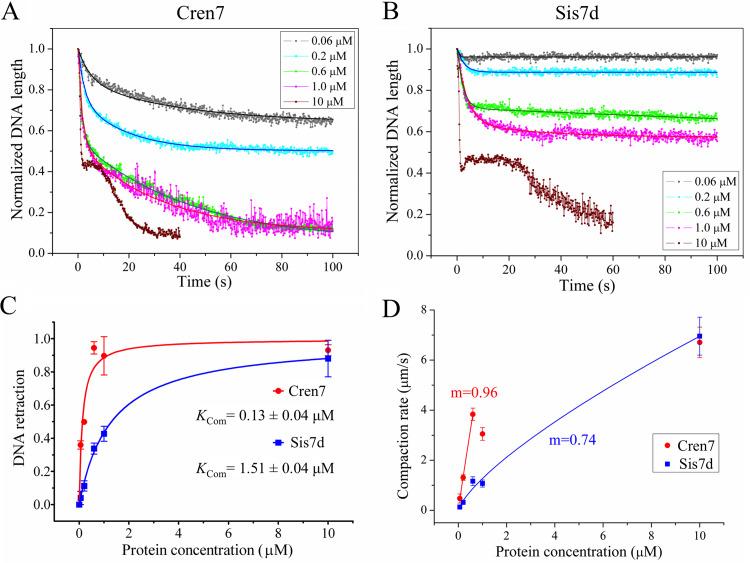
Kinetic analysis of DNA compaction by Cren7 and Sis7d. (A and B) DNA retraction (shown in dots) was induced by Cren7 (A) and Sis7d (B) at different concentrations. Each data set represents averages of the measurements of 20 DNA molecules. A second-order exponential decay fit for each data set is shown as a solid line. (C) Reductions in normalized DNA length (-ΔL) as a result of binding by Cren7 and Sis7d are plotted against protein concentrations. The data points were fitted using the following equation: Δ*L* = Δ*L*_max_ * *C*/ (*K*_Com_ + *C*), where Δ*L*_max_ is the maximum change in DNA length, *C* is the protein concentration, and *K*_Com_ is the apparent-compaction constant. The calculated *K*_Com_ values are 0.13 ± 0.04 μM for Cren7 and 1.51 ± 0.09 μM for Sis7d, with *R*^2^ values of 0.962 and 0.998, respectively. (D) The rates of initial compaction by Cren7 and Sis7d are plotted against protein concentrations. A power law fit to the initial data points for each protein is shown in solid line. Best-fit exponents (m) are 0.96 for Cren7 and 0.74 for Sis7d, with *R*^2^ values of 0.999 and 0.996, respectively.

To determine the kinetics of DNA compaction by Cren7 and Sis7d, single-molecule experiments were conducted at various protein concentrations, and ∼20 DNA molecules were monitored in each experiment. The average time courses of the size change of the DNA were obtained for Cren7 and Sis7d at specified concentrations. As shown in Fig. 2A and B, both proteins elicited single-step DNA compaction at all tested concentrations except for 10 μM. The best fit for the single-step curves was described by a second-order exponential decay function. The fit agrees well with a two-state model, which entails 1:1 (ligand/binding site) binding of the protein to the immobilized DNA followed by a conformational change of the resulting complex. The final length of the DNA after compaction (*L*_f_) is derived from each fitting curve, and the packing ratio, defined as the ratio of the contour length of the DNA before the compaction to that after the compaction, is therefore the reciprocal of *L*_f_ (Table 1). The packing ratios for both proteins increased with increasing protein concentrations. The maximum packing ratio of ∼10 for Sis7d was obtained at the protein concentration of 10 μM. By comparison, the DNA packing ratio for Cren7 reached 10 at 0.6 μM. At higher protein concentrations, Cren7 further condensed the DNA to a final length of ∼1 μm, which is close to the resolving limit of resolution of fluorescence microscopy. Given the initial length of the DNA (∼13 μm), we suggest that the packing ratio of Cren7 was greater than 13. We then calculated the apparent compaction constants (*K*_Com_), defined as the protein concentration required to achieve half-maximal DNA compaction at equilibrium, of the two proteins. As shown in Fig. 2C, the *K*_Com_ values for Cren7 and Sis7d were 0.13 ± 0.04 and 1.51 ± 0.09 μM, respectively, indicating that Cren7 was ∼12-fold more efficient than Sis7d in DNA compaction. Notably, the *K*_Com_ of Cren7 is very close to the apparent dissociation constant (*K_D_*) values of dsDNA binding by the protein (0.126 μM) obtained under identical conditions (Fig. S3). Therefore, DNA compaction correlates directly with DNA binding by Cren7. On the other hand, the *K*_Com_ of Sis7d is about 2-fold higher than its *K_D_* (0.780 μM) (Fig. S3), suggesting that more Sis7d protomers than those bound to DNA may be required for DNA compaction.

**TABLE 1:** Kinetic parameters of DNA compaction for Sis7d and Cren7

Protein and concn (μM)	Compaction rate *v_i_* (μm/s)^*a*^	Packing ratio^*b*^
Sis7d		
0.06	0.13 ± 0.04	1.0
0.2	0.31 ± 0.02	1.1
0.6	1.16 ± 0.17	1.5
1.0	1.07 ± 0.14	1.7
10	6.96 ± 0.76	>10
		
Cren7		
0.06	0.47 ± 0.18	1.6
0.2	1.31 ± 0.11	2.0
0.6	3.84 ± 0.25	10
1.0	3.05 ± 0.26	>10
10	6.71 ± 0.61	>10

a The compaction rate (*v_i_*) data shown here refer to the rates of the initial stage (the first 1 to 1.5 s) of the DNA retraction trajectory. The mean initial lengths of λ DNA molecules in the experiments performed for Sis7d and Cren7 were 13.22 and 12.98 μm, respectively.

b The packing ratios of the DNAs, which were finally condensed into a dot of ∼1-μm diameter, were all defined as >10.

10.1128/mBio.00804-20.4 FIG S3 Kinetic characterization of the binding of Cren7 (A) and Sis7d (B) to short dsDNA fragments by SPR. Cren7 or Sis7d was injected over immobilized DNAs. Sensorgrams are shown with the protein concentrations labeled. RU, response units. Equilibrium and kinetic constants were calculated using BIA evaluation 4.1 software by a global fit to a 1:1 Langmuir binding model for Cren7 (A) and a steady-state binding model for Sis7d (C). Download FIG S3, JPG file, 0.7 MB. Copyright © 2020 Zhang et al. 2020 Zhang et al. This content is distributed under the terms of the Creative Commons Attribution 4.0 International license.

Intriguingly, the assembled protein-DNA complexes were not readily decompacted since washing with the imaging buffer did not lead to measurable changes in the contour length of DNA condensed by Cren7 or Sis7d within a time period of up to 10 min in this study (data not shown). This is consistent with a recent finding that the Cren7-DNA and Sis7d-DNA complexes were stable for up to 10 min even at an extension force of 3.5 pN (20). Therefore, we were able to measure only the rates of DNA compaction by the proteins but not the decompaction rates of the protein-DNA complexes. As shown in Fig. 2A and B, the compaction rates dropped rapidly in the initial phase (i.e., the first 2 s) of DNA compaction. The initial compaction rate (v_i_, in μm/s) for Cren7 or Sis7d was defined as the steepest slope of the initial phase of the curve at an indicated concentration. As shown in Table 1, at each tested concentration between 0.06 and 1.0 μM, the initial compaction rate of Cren7 was nearly 4-fold higher than that of Sis7d, in agreement with higher affinity of Cren7 than of Sis7d for DNA. At the saturating concentration (i.e., 10 μM), however, the initial compaction rates for Cren7 and Sis7d (6.71 ± 0.61 and 6.96 ± 0.76 μm/s, respectively) were nearly identical, suggesting similar patterns of the two proteins in the initial phase of DNA compaction. In addition, the initial rates of DNA compaction by both proteins increased almost linearly with protein concentration before the level of saturation was reached (Fig. 2D), as expected from the lack of cooperativity in DNA binding and distortion by the two proteins (5, 22).

### Cren7 and Sis7d compact DNA in a three-step process at high protein concentrations.

When Cren7 or Sis7d was injected at 10 μM, the DNA was retracted in three discrete steps, i.e., two compaction steps and an intervening noncompaction step (Fig. 2A and B). The two compaction steps proceeded at distinct rates, pointing to the differences in the modes of compaction. In the first compaction step, Cren7 and Sis7d compacted DNA in similar fashions. Both proteins shortened the DNA molecule by nearly 60% in our assays where the DNA was under an estimated extension force of approximately 0.2 to 0.3 pN. This finding agrees with the previous observations made by using magnetic tweezers that Cren7 and Sul7d reduced the length of DNA by ∼50% at an extension force of 0.5 pN (20). The crystal structures of DNA bound by multiple Cren7 molecules revealed no changes in DNA length (21). However, it was shown previously that DNA bending induced by Cren7 and Sul7d reduced the persistence length of DNA in solution (20), presumably allowing DNA retraction at low extension forces. Therefore, it appears that the increased flexibility of DNA, as a result of the bending by Cren7 and Sis7d, played a major part in the first DNA compaction step. In the noncompaction step, referred to as the lag step, the end-to-end length of the DNA remained largely unchanged. Therefore, we speculate that, once the DNA was progressively bound, in a side-by-side fashion, to saturation in the first DNA compaction step, the protein-DNA complex underwent rearrangement in the lag step, which was required for the further condensation of the DNA. Interestingly, the lag step in DNA compaction by Sis7d was much longer than that by Cren7 (23 s versus 7 s), indicating that the architectural rearrangement of the Sis7d-DNA complex was more complex than that of the Cren7-DNA complex. During the second compaction step, λ DNA molecules were further retracted to the tether point to form condensed globular structures characteristic of the consequences of DNA bridging. The notion that DNA bridging is involved in DNA compaction by Cren7 and Sis7d agrees with previous observations in similar single-molecule studies indicating that DNA bridging proteins (24–26), but not DNA bending proteins (26–28), compact flow-stretched DNAs to the tether point to form a condensed globular structure. The rates of DNA compaction in this step were much lower than those in the bending step for both Cren7 and Sis7d. Notably, Cren7 was more efficient than Sis7d in DNA bridging since the rate of compaction by the former (0.32 ± 0.02 μm/s) was significantly higher than that by the latter (0.13 ± 0.03 μm/s) in this step. On the basis of these data, we suggest that Cren7 and Sis7d compact DNA through DNA bending at lower protein/DNA ratios and through DNA bridging at higher protein/DNA ratios.

**FIG 3:**
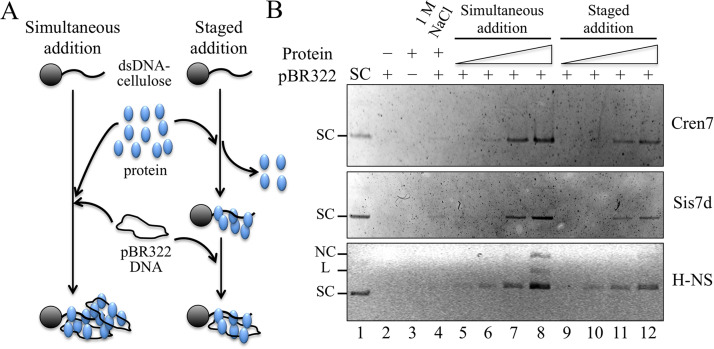
DNA bridging by Cren7 and Sis7d. (A) A diagram showing dsDNA-cellulose pulldown assays. Double-stranded DNA−cellulose was incubated with Cren7 or Sis7d as well as pBR322 DNA. Unbound protein and pBR322 DNA were removed by centrifugation. (Left panel) The protein and pBR322 DNA were mixed simultaneously with DNA-cellulose. (Right panel) The protein was added to DNA-cellulose first, unbound protein was removed by centrifugation, and pBR322 DNA was then added. (B) Detection of pBR322 DNA associated with DNA-cellulose by agarose gel electrophoresis. The dsDNA-cellulose pulldown assays were performed as described for panel A. After removal of unbound protein and pBR322 DNA by centrifugation, the pellet was resuspended in elution buffer. The bound pBR322 DNA was eluted with elution buffer. The eluted DNA was subjected to agarose gel electrophoresis. H-NS from E. coli (EcH-NS) was used as a positive control. Aliquots of the eluates (20 μl for each Cren7/Sis7d sample and 10 μl for each EcH-NS sample) were loaded. Lane 1, supercoiled pBR322 DNA; lane 2, protein omitted; lane 3, pBR322 DNA omitted; lane 4, the assay was carried out in the presence of 1 M NaCl; lanes 5 to 8, protein (0, 0.3, 1, or 10 μM) and pBR322 DNA (250 ng) were mixed simultaneously with DNA-cellulose (∼250 ng DNA); lanes 9 to 12, protein (0, 0.3, 1, or 10 μM) was added to DNA-cellulose (∼250 ng DNA), unbound protein was removed by centrifugation, and pBR322 DNA (250 ng) was then added. Bands corresponding to linear (L), supercoiled (SC), or nicked circular (NC) pBR322 DNA are indicated. The presence of linear and nicked circular pBR322 DNA in the H-NS samples was probably due to the nuclease contamination in the protein preparation.

To further test the ability of Cren7 and Sis7d to bridge DNA, we performed pulldown assays (29, 30). A mixture of dsDNA-cellulose and pBR322 DNA (4,361 bp) was incubated with Cren7 or Sis7d, and, after washing, pBR322 DNA associated with the DNA-cellulose was recovered and quantitated (Fig. 3). As shown in Fig. 3B, pBR322 DNA was recovered along with the DNA-cellulose in the presence of either Cren7 or Sis7d. Addition of 1 M NaCl to the mixture led to a substantial decrease in the amount of recovered plasmid DNA. This observation is consistent with the notion that the two proteins mediate DNA bridging. The amount of pBR322 DNA recovered increased with increasing concentrations of the protein. Cren7 appeared to be more efficient than Sis7d in bridging DNA. It is worth noting that, when Cren7 or Sis7d was mixed with DNA cellulose and then washed prior to the addition of pBR322 DNA, less DNA-cellulose-associated plasmid DNA was obtained (compare lanes 5 to 8 with lanes 9 to 12 in Fig. 3B). Therefore, in agreement with the SM-TIRF results, both Cren7 and Sis7d were able to mediate DNA bridging and this effect was most pronounced in the presence of excess amounts of the protein.

### Oligomerization of Cren7 and Sis7d.

**FIG 4:**
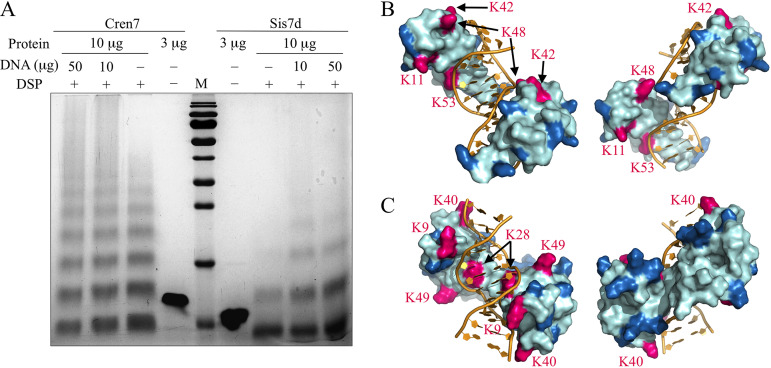
Chemical cross-linking of Cren7 and Sis7d. (A) Cren7 or Sis7d was cross-linked in the absence or presence of pUC18 DNA with 1 mM DSP. After incubation for 30 min at 25°C, reactions were terminated and analyzed by SDS-PAGE. (B and C) Structural models of a complex of two Cren7 molecules with a 12-bp dsDNA (B) and a complex of two Sis7d molecules with a 12-bp dsDNA (C) were constructed by using the crystal structure of Cren7 in complex with an 18-bp DNA (PDB code: 6A2I) and the solution structure of Sso7d in complex with a 12-bp DNA (PDB code: 1BBX) as the templates, respectively. Lysine residues involved in intermolecular cross-linking are shown in red and labeled, and the remaining lysine residues are shown in blue.

A chromatin protein may mediate DNA bridging through protein-protein interactions. To investigate the potential role of Cren7 and Sis7d in DNA bridging, we first examined the intermolecular contacts between Cren7 molecules and between Sis7d molecules by chemical cross-linking using dithiobis (succinimidyl propionate) (DSP), a 1.2-nm cross-linker reactive toward amino groups in protein (Fig. 4). We found that Cren7 was readily cross-linked into dimers, trimers, and larger oligomers in the absence of DNA. The presence of DNA at protein/DNA mass ratios of 1:1 and 1:5 significantly increased the cross-linking efficiency and thus the formation of larger polymers, suggesting that Cren7 more readily forms protein clusters in a DNA-bound form than in solution. In comparison, Sis7d was cross-linked much less efficiently than Cren7, forming predominantly dimers in the absence of DNA. And again, the efficiency of cross-linking of Sis7d into dimers and even larger oligomers was enhanced in the presence of DNA at the protein/DNA mass ratios of 1:1 and 1:5. It appears that DNA binding promotes the oligomerization of Cren7 and Sis7d in solution. These results are consistent with the observation in our single-molecule experiments that the two proteins facilitated higher-order bridging of λ DNA strands. The difference between Sis7d and Cren7 in their abilities to oligomerize also provides an explanation for the difference between the two proteins in their efficiencies in compacting DNA into highly condensed structures.

To learn more about the structural basis of DNA bridging by Cren7 and Sis7d, we cross-linked each of the proteins with disuccinimidyl suberate (DSS) (a 1.14-nm cross-linker reactive toward amino groups in protein) and subjected the cross-linking products to digestion with trypsin and the digests to nano-liquid chromatography–tandem mass spectrometry (nano-LC-MS/MS) analysis. A large number of cross-linked peptides were identified, and most of the lysine residues of each protein were found to be involved in the cross-linking (Table 1). Since both Cren7 and Sis7d are lysine-rich proteins, intramolecular cross-linking is presumably responsible for a significant fraction of the detected cross-linking products containing different peptide sequences, as suggested by the distance between the two cross-linked lysine residues. Therefore, we considered only two cross-linked peptides containing the same lysine residue to be the intermolecular cross-linking sites (Fig. S4; see also Fig. S5). For each protein, four lysine residues (i.e., residues K11, K42, K48, and K53 in Cren7 and residues K9, K28, K40, and K49 in Sis7d) were found to be involved in the intermolecular cross-linking (Fig. 4B and C). Modeling of these sites on the structures of the Cren7-DNA complex, derived from the crystal structure of Cren7 in complex with 18-bp DNA (21), and of the Sis7d-DNA complex, derived from the solution structure of Sso7d in complex with a 12-bp DNA (23), revealed that these sites were not involved in the lateral interaction between two neighboring Cren7 or Sis7d molecules bound to the same DNA duplex, given the distance between the same residues from the two protein (Fig. 4B and C). Therefore, the presence of the four sites indicates that both Cren7 and Sis7d are capable of bridging DNA duplexes through interactions between DNA-bound protein molecules. Notably, the intermolecular cross-linking sites from the two adjacent Cren7 molecules bound to the DNA point in opposite directions whereas those from the two adjacent Sis7d molecules on the DNA were located on the same side. Sterically, therefore, the Cren7-DNA complexes readily formed higher-order structures via protein-protein interactions. On the other hand, the Sis7d-DNA complexes were less flexible in cross-linking into higher-order structures. Taken together, our data indicate that both Cren7 and Sis7d are able to mediate DNA bridging though protein-protein interactions, albeit with different levels of efficiency.

10.1128/mBio.00804-20.5 FIG S4 MS/MS spectra of interprotein crosslinked peptides for Cren7. (A) DPETGKYFR-DPETGKYFR; (B) HKLPDDYPI-HKLPDDYPI; (C) IGLFKDPETGK-IGLFKDPETGK; (D) VKTPAGK-VKTPAGK. Sites of crosslinking are underlined. Peaks in the MS and MS/MS spectra matched well with the theoretical precursor and sequence ions. Download FIG S4, JPG file, 1.2 MB. Copyright © 2020 Zhang et al. 2020 Zhang et al. This content is distributed under the terms of the Creative Commons Attribution 4.0 International license.

10.1128/mBio.00804-20.6 FIG S5 MS/MS spectra of interprotein crosslinked peptides for Sis7d. (A) GAVSEKDAPK-GAVSEKDAPK; (B) MISFTYDEGGGKTGR-MISFTYDEGGGKTGR; (C) VGKMISFTYDEGGGK-VGKMISFTYDEGGGK; (D) YKGEEK-YKGEEK. Crosslinking sites are underlined. These MS/MS spectra demonstrated the interprotein crosslinking, providing robust evidence for the protein oligomerization. Peaks in the experimental MS and MS/MS spectra matched well with the theoretical precursor and sequence ions. Download FIG S5, JPG file, 1.4 MB. Copyright © 2020 Zhang et al. 2020 Zhang et al. This content is distributed under the terms of the Creative Commons Attribution 4.0 International license.

### Cren7 and Sis7d form higher-order structures on DNA.

**FIG 5:**
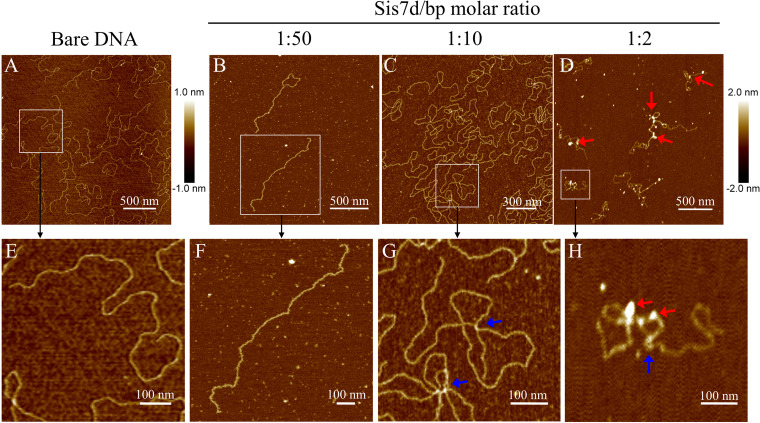
AFM images of cross-linked Sis7d-DNA complexes. Linear pBR322 DNA was incubated with Sis7d at protein/DNA ratios (in protomers/base pair) of 0 (A and E), 1:50 (B and F), 1:10 (C and G), and 1:2 (D and H). Sites of DNA bridging and clustering are indicated by blue and red arrows, respectively.

To gain more insights into the architectural roles of Cren7 and Sis7d in DNA compaction, we mixed linear pBR322 DNA with Cren7 or Sis7d and visualized the complexes by the use of AFM (Fig. S6). Both proteins induced DNA compaction at protein/DNA ratios of 1:10 (one protein monomer to 10 bp DNA) and 1:2, in agreement with the previous finding (20). However, the protein-DNA complexes formed at higher protein/DNA ratios (e.g., 1:0.4) were difficult to observe because of the presence of layers of unbound protein attached to the mica surface. Subsequently, we first cross-linked complexes of Cren7 or Sis7d with linear pBR322 DNA with glutaraldehyde and then observed the cross-linked products under AFM. The conformation of the naked pBR322 DNA was not affected by treatment with glutaraldehyde, and the mean contour length of the DNA was ∼1,498 nm, which was slightly longer than the theoretical length of the DNA (1,483 nm). Sis7d molecules were sparsely distributed along the DNA filament, and few changes in the conformation of the bound DNA were observed at the protein/DNA ratio of 1:50 (Fig. 5B and F). DNA bridging became apparent at the protein/DNA ratio of 1:10, as intramolecular cross-links were observed on nearly all protein-DNA complexes (Fig. 5C and G). Sis7d formed clusters on the DNA when the protein/DNA ratio further increased to 1:2 (Fig. 5D and H). The sizes and numbers of the clusters on each DNA molecule differed. However, the contour length of the Sis7d-DNA filament was significantly shorter (∼1,302 nm) than that of the naked DNA, raising the possibility that the DNA sequences in the clusters were compacted.

10.1128/mBio.00804-20.7 FIG S6 AFM images of Sis7d-DNA and Cren7-DNA complexes. Linear pBR322 DNA was incubated for 30 min with Sis7d or Cren7 at the protein/DNA ratios (in monomers/base pair) of 0, 1:50, 1:10, 1:2, and 1:0.4. The scan size of each image is labeled. Download FIG S6, JPG file, 2.2 MB. Copyright © 2020 Zhang et al. 2020 Zhang et al. This content is distributed under the terms of the Creative Commons Attribution 4.0 International license.

**FIG 6:**
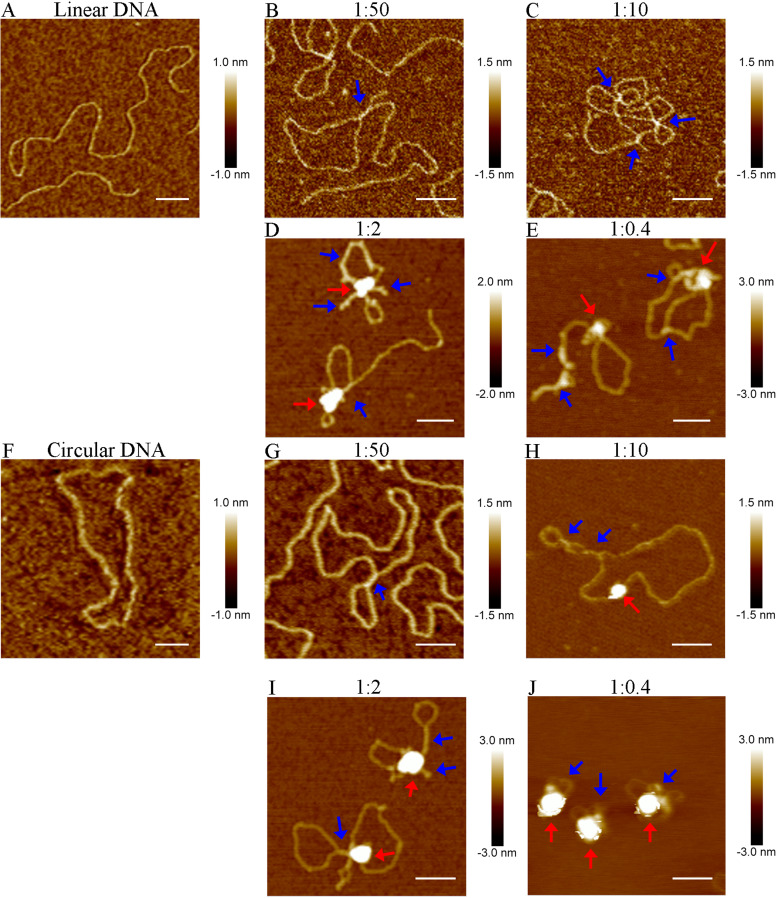
AFM images of individual cross-linked Cren7-DNA complexes. Linear pBR322 DNA (A to E) or singly nicked pBR322 DNA (F to J) was incubated with Cren7 at protein/DNA ratios (in protomers/base pair) of 0 (A and F), 1:50 (B and G), 1:10 (C and H), 1:2 (D and I), and 1:0.4 (E and J). Sites of DNA bridging and condensations are indicated by blue and red arrows, respectively. Scale bar represents 100 nm.

Cren7 induced DNA bridging in a manner similar to that seen with Sis7d but did so more efficiently and condensed DNA differently (Fig. S7). DNA bridging by Cren7 was found in ∼95% of the protein-DNA complexes even at the protein/DNA ratio of 1:50 (Fig. 6B). Multiple cross-links and loops were readily formed in a single DNA molecule by the protein at the protein/DNA ratio of 1:10 (Fig. 6C). The DNA became even more highly compacted with a further increase in the protein/DNA ratio (Fig. 6D). The majority (∼85%) of the Cren7-DNA complexes formed at the protein/DNA ratio of 1:2 contained only a single extremely condensed core-like structure, from which the uncondensed DNA segments were seen to be spreading out as loops and filaments. This contrasts with the random distribution of multiple clusters on a Sis7d-bound DNA molecule. At higher protein/DNA ratios, the compact core became larger and the uncondensed spreading DNA segments were shorter (Fig. 6E). It was noticed that most (∼65%) of the Cren7-DNA complexes assembled at the protein/DNA ratio of 1:0.4 showed only a single free DNA end and that the remainder had no free ends (∼25%) or two free ends (10%) (Fig. S7E). To test whether the free ends of DNA are required for DNA condensation by Cren7, relaxed circular pBR322 DNA containing a single nick was used in the cross-linking assays. We found that the circular DNA was more efficiently compacted by Cren7 than the linear DNA (Fig. 6F to J). For example, highly condensed core-like structures were found even at the low protein/DNA ratio of 1:10 (Fig. 6H). It appears possible that the interaction between DNA-bound protein molecules would be enhanced on a circular DNA compared to that on a linear DNA. Like linear DNA, circular DNA was condensed by Cren7 to form only a single condensed core (Fig. 6H to J). On the basis of these results, we conclude that Cren7 and Sis7d were able to assemble distinct higher-order structures upon binding to DNA, which are suggestive of the different architectural roles of the two proteins, and that intramolecular bridging of DNA strands was involved in the assembly process.

10.1128/mBio.00804-20.8 FIG S7 AFM images of crosslinked Cren7-DNA complexes. Linear pBR322 DNA (A to E) or singly nicked pBR322 DNA (F to J) was incubated with Cren7 at the protein/DNA ratios (in monomers/base pair) of 0 (A and F), 1:50 (B and G), 1:10 (C and H), 1:2 (D and I), and 1:0.4 (E and J). Sites of DNA bridging and condensations are indicated by blue and red arrows, respectively. The scan size of each image is labeled. Download FIG S7, JPG file, 2.6 MB. Copyright © 2020 Zhang et al. 2020 Zhang et al. This content is distributed under the terms of the Creative Commons Attribution 4.0 International license.

## DISCUSSION

Cren7 and Sul7d, the two major chromatin proteins from *Sulfolobus* species, are similar in structure and share biochemical properties such as the ability to bend DNA and to constrain DNA supercoils *in vitro* (5, 14). In this report, by using a single-molecule approach, we show that the two proteins were able to compact DNA. While Cren7 was more efficient than Sis7d in DNA compaction, both proteins were able to compact DNA into a highly condensed globular structure at saturation, suggesting that DNA bridging was involved in the process. Further analyses revealed that the two proteins behaved similarly in DNA bending but differently in DNA bridging: Sis7d formed discrete clusters whereas Cren7 generated a single highly condensed core on the test DNA fragment.

Our single-molecule experiments revealed that both Cren7 and Sis7d compacted a flow-stretched DNA to the tether point to form a condensed globular structure. This property has been shown to be characteristic of DNA bridging proteins, such as ParB and SMC (24, 25). On the other hand, DNA bending proteins are unable to do so since each bending event reduces the DNA length by only a modest fraction (26–28). A single-molecule study using magnetic tweezers showed that both Cren7 and Sis7d compacted DNA by reducing the length of the target DNA by ∼50% at an extension force of 0.5 pN (20). The discrepancy between their finding and ours probably results from the ways in which the target DNA molecule was tethered. The DNA molecule was singly tethered at one end in our study but doubly tethered at both ends in theirs. The DNA molecules were subjected to similar extension force (at the sub-pN level) in both cases. However, tension experienced by the singly tethered DNA molecule would decrease along its length and eventually drop to zero at the free end of the DNA in our experimental system, whereas that experienced by the doubly tethered DNA would be rather constant. Since DNA loop formation is sensitive to applied force, DNA toward its free end, where the tension is the lowest, would be more readily compacted by DNA bridging proteins (31). Therefore, the singly tethered DNA was more sensitive than the doubly tethered DNA to DNA bridging and compaction by Cren7 and Sis7d.

DNA compaction by both Cren7 and Sis7d at high concentrations (e.g., 10 μM) appeared to proceed in three steps: the initial bending step and the final bridging step separated by a lag step of conformational change. In the initial bending step, the two proteins compacted DNA at similar rates and shortened DNA by ∼60%, a level comparable to those reported by others from experiments performed using magnetic tweezers (20), supporting the notion that DNA retraction at this step is caused primarily by DNA bending. However, Cren7 induced DNA bridging more efficiently than Sis7d. This is consistent with the greater ability of the former than the latter to form higher-order structures on DNA. The presence of the lag step suggests that DNA bridging by Cren7 or Sis7d occurred in an ordered fashion. AFM images of the cross-linked protein-DNA complexes formed at various protein/DNA ratios shed light on the entire process of DNA organization by Cren7 and Sis7d, including DNA binding, bridging, and condensation. The binding site size has been estimated to be ∼6 bp per monomer for Cren7 and ∼4 bp per monomer for Sso7d (23, 32). Cren7 started to bridge DNA even at the protein/DNA ratio of one monomer to 50 bp. Multiple sites of bridging were apparent at the protein/DNA ratio of 1:10, when the DNA was nearly completely coated by the protein. DNA was further condensed by Cren7 to form a core-like structure at the saturating protein/DNA ratio of 1:2. The condensed core-like protein-DNA complexes, especially those formed on the circular DNA, were uniform in appearance and surrounded by DNA loops. In contrast, Sis7d was less efficient than Cren7 in generating intramolecular cross-links and formed randomly distributed protein-DNA clusters on DNA at the saturating protein/DNA ratios.

Mechanistically, protein-induced DNA compaction via bending and bridging may result from the cooperativity of the interaction between proteins which bend or elastically deform the DNA or from DNA looping through protein-mediated DNA bridging, as demonstrated in theoretical models (37, 38, 39). Both Cren7 and Sis7d were able to induce rigid bends in DNA and increase its elasticity by reducing the apparent persistence length (20). The resulting elastic deformation in different stretches of DNA would facilitate clustering of the proteins and thus generate deformed regions, leading to DNA compaction. On the other hand, both proteins formed oligomers, mediating DNA bridging. Local bridging would increase DNA concentration, which would in turn further enhance DNA binding and bridging, possibly leading to phase separation in a single DNA molecule with the formation of condensed cores along with DNA loops, as most clearly shown in the case of Cren7, which was more efficient in bridging DNA than Sis7d.

By estimation, the genomic DNA is fully coated by Cren7 and Sul7d in the *Sulfolobus* cell. The two proteins may not be distributed evenly on the genome because Cren7 is known to bind preferentially to AT-rich DNA sequences whereas Sso7d binds DNA without apparent sequence specificity (21, 22). The possibility exists that Sul7d forms clusters on the DNA and Cren7 induces DNA condensation by cross-linking DNA at multiple sites. Other *Sulfolobus* chromatin proteins are also expected to cooperate with Cren7 and Sul7d in chromosomal DNA organization. For instance, Sso10a1 and Sso10a2, two Sso10a paralogues from S. solfataricus, are able to bridge and stiffen DNA duplexes, respectively (33). It appears that interplay among various chromatin proteins serving distinct architectural roles dynamically shapes the crenarchaeal genomic DNA.

## MATERIALS AND METHODS

### Protein overproduction and purification.

Recombinant Cren7 and Sis7d were overproduced and purified as described previously (17, 34). Protein concentrations were determined by the Lowry method using bovine serum albumin (BSA) as the standard.

### Preparation of biotin-labeled dsDNA fragments.

Oligonucleotides, synthesized commercially at Sangon BioTech (Shanghai, China), were dissolved in 10 mM HEPES-KOH (pH 7.6)–100 mM KCl. Biotinylated λ DNA was prepared by annealing a 3′-biotinylated oligonucleotide (5′-AGGTCGCCGCCTEG-biotin-3′) and an unlabeled oligonucleotide (5′-GGGCGGCGACCT-3′) to the cohesive ends of λ DNA and was ligated with T4 DNA ligase (NEB). Biotin-labeled DNA fragments used in surface plasmon resonance (SPR) assays were made by annealing indicated oligonucleotides.

### Single-molecule visualization of DNA compaction.

The single-molecule visualization of DNA compaction was carried out as described previously (35) with modifications. Flow cell chambers were assembled using a glass slide, a coverslip, and double-sided tape. In brief, the coverslips were amino functionalized with 1% (vol/vol) aminopropyltriethoxysilane (Sigma)–methanol/acetic acid (20:1) (Sigma) at room temperature and were then biotinylated by incubation in a mixture of mPEG-SVA-2000 and biotin-PEG-SVA-2000 (Lysan Bio. Inc.) at a molar ratio of 1:25. The biotinylated λ DNA (1.5 nM) was immobilized onto the biotin functionalized surface of the flow cell through the interaction with streptavidin. Free DNA was washed out from the flow cell with imaging buffer (10 mM HEPES-KOH [pH 7.6], 100 mM KCl, 2.5 mM MgCl_2_, 0.1 mg/ml bovine serum albumin [BSA]). Tethered DNA was stained with Sytox orange fluorescent dye (Thermo Fisher Scientific) at a concentration of 10 nM in imaging buffer. The flow rate was set at 100 μl/min, using an aspiring syringe pump (Harvard Apparatus, standard infuse/withdraw Pump 11 Elite), to stretch out singly tethered DNA molecules. Under our experimental conditions, the extension force of the singly tethered DNA was estimated to be at the subpiconewton level based on the simulation reported previously (36). Cren7 or Sis7d samples were pumped into the flow cell at 100 μl/min. A time-lapse movie of the behavior of the immobilized DNA molecules for each experiment was acquired by an inverted total internal reflection fluorescence microscope (modified from IX-71; Olympus) (60× lens objective with numerical aperture [NA] = 1.49) with an Andor iXon DU897 electron-multiplying-charge-coupled-device (EMCCD) camera at 0.1-s exposure and 0.1-s intervals for 700 frames.

### Data analysis.

The initial and final lengths of ∼30 DNA molecules in each experiment were measured manually using ImageJ Ver1.33u software (Wayne Rasband, NIH, USA). The mean length was obtained by Gaussian fitting. To determine the kinetics of DNA compaction induced by Cren7 and Sis7d, real-time changes in DNA length were monitored using Matlab 2016a as previously described (35). For each experiment, measurements of over 20 individual DNA molecules were obtained after removing short, aberrant, and stuck DNA molecules. Changes in the normalized length of these DNA molecules were plotted against time, and the results were averaged to yield a kinetic curve. The rate of DNA compaction by Cren7 or Sis7d (in μm/s) was derived from the slope of the DNA shortening trajectory, and the packing ratio was obtained by dividing the initial length by the final length of the DNA molecule in a compaction event.

### Surface plasmon resonance (SPR) assays.

SPR assays were performed at 25°C on a BIAcore T100 instrument (BIAcore AB, Uppsala, Sweden). The running buffer was identical to the imaging buffer used in the single-molecule experiments except for the supplementation of 0.005% (vol/vol) Tween 20. The biotin-labeled dsDNA fragment (5′-biotin-TTTCTACCCTTTGGTGCTAATGCCCATACT) was captured on the SA sensor chip (91 to 97 response units). A blank flow cell was used to correct for instrumental and concentration effects. Cren7 or Sis7d, at a concentration in a range spanning the *K_D_* of the binding of the protein to dsDNA, was injected over the DNA surface and the blank flow cell for 2 min at a flow rate of 30 ml/min. After the dissociation phase (2 to 4 min), the bound protein was removed with a 30-s wash with 0.01% SDS, followed by a 60-s buffer injection. The measurement was repeated once at the lowest protein concentration for each experiment. Equilibrium and kinetic constants were calculated by a global fit to a 1:1 Langmuir binding model or steady-state binding model (BIA evaluation 4.1 software).

### DNA-cellulose pulldown assays.

Pulldown assays were conducted as described previously (29) with modifications. DNA-cellulose (Sigma-Aldrich; ∼5 mg double-stranded calf thymus DNA/g), instead of the streptavidin-coated Dynabeads coupling the biotinylated duplex DNA fragments, was used in the assays. DNA-cellulose was washed twice with washing buffer (10 mM Tris-Cl [pH 7.5], 2 M NaCl, 5 mM EDTA, 0.1 mg/ml BSA) and twice with imaging buffer (10 mM HEPES-KOH [pH 7.6], 100 mM KCl, 2.5 mM MgCl_2_, 0.1 mg/ml BSA). For each pulldown experiment, 0.05 mg DNA-cellulose, containing ∼250 ng dsDNA, was resuspended in 50 μl of imaging buffer containing indicated amount of Cren7 or Sis7d (0, 15, 50, or 500 pmol, or 0, 0.3, 1, or 10 μM) and 250 ng pBR322 DNA (∼84 fmol). After incubation for 30 min at room temperature, the samples were washed twice with 50 μl of imaging buffer, and the bound pBR322 DNA was eluted with 50 μl of elution buffer (10 mM Tris-Cl [pH 7.5], 5 mM EDTA, 200 mM NaCl, 0.2% SDS, 0.5 mg/ml proteinase K). The eluted DNA was resolved by gel electrophoresis through a 1% agarose gel, stained with SYBR gold (Molecular Probes), and imaged using a Tanon 4200 multi-imaging system (Tanon, China). Escherichia coli H-NS protein (EcH-NS) was used as a positive control (30).

### Chemical cross-linking.

Cren7 or Sis7d (10 μg) was cross-linked with 1 mM dithiobis (succinimidyl propionate) (DSP) for 30 min at 25°C in 20 mM HEPES-KOH (pH 7.6)–50 mM KCl in the presence or absence of pUC18 DNA (10 or 50 μg, respectively) in a final volume of 10 μl. Cross-linking reactions were stopped by the addition of 50 mM Tris-HCl (pH 7.5). Samples were mixed with an equal volume of 2× loading buffer (omitting β-mecaptoethanol) and subjected to 15% SDS-PAGE. Gels were stained with Coomassie brilliant blue R-250.

### Nano-LC-MS/MS analysis of chemically cross-linked proteins.

Cren7 or Sis7d was dissolved in 20 μl of 50 mM HEPES-KOH (pH 7.8)–150 mM NaCl to reach a final concentration of 0.5 mg/ml. Following the addition of 2 μl of the cross-linker disuccinimidyl suberate (DSS) solution (dissolved in dimethyl sulfoxide [DMSO]), the mixtures were incubated for 1 h at room temperature. The reaction was terminated by addition of 0.9 μl of 500 mM ammonium bicarbonate and incubation for 20 min. The cross-linked proteins were precipitated with acetone and collected by centrifugation. The samples were digested with trypsin, and the tryptic peptides were desalted using ZipTip C18 before nano-LC-MS/MS analysis. The peptide samples were analyzed using an Exactive Plus Orbitrap mass spectrometer coupled with an EASY-nLC 1200 system (Thermo Fisher Scientific, Rockford, IL, USA). Peptide separation was carried out on a homemade C_18_ column (15 cm by 75 μm, 3-μm pore size, 100 Å) at a flow rate of 0.5 μl/min. Peptides were separated using a 100-min linear gradient ranging from 5% to 80% phase B (mobile phase A, 0.1% formic acid [FA]; mobile phase B, 0.1% FA–acetonitrile [ACN]). Data-dependent acquisition was performed. The MS survey was performed in the Fourier transform (FT) cell with a mass range of 350 to 2,000 *m*/*z*. Meanwhile, the resolution was set as 70,000, and the automatic gain control (AGC) was set to 3,000,000 ions. For MS/MS analysis via high-energy collisional dissociation (HCD), a resolution of 7,500 was used in the Orbitrap analyzer with an isolation window of 1.8 *m*/*z*, a target value of 50,000 ions, and maximum accumulation time of 50 ms. Fragmentation was performed with normalized collision energies of 27%, and the 20 most intense signals in the survey scan were fragmented. Dynamic exclusion duration was set as 40 s, and the minimum MS signal for triggering MS/MS was set to 5,000. Two technical repetitions were performed. The MS raw data were searched against the protein sequence downloaded from Uniprot using pLink 2.0. The parameters were set at default as follows: precursor and fragment tolerance, 20 ppm; up to 3 missed cleavages; oxidation in M as variable modification.

### Atomic force microscopy (AFM).

Linear and nicked plasmid pBR322 DNAs were prepared by treatment with EcoRI and Nb.Bpu10I, respectively, and were purified by using a PCR clean-up system (Promega Co., Madison, WI, USA). Cren7 or Sis7d was incubated for 40 min at room temperature with the DNA (100 ng) at a monomer/base pair ratio of 1:50, 1:10, 1:2, or 1:0.4 in imaging buffer (excluding BSA) in a final volume of 20 μl. Aliquots (5 μl) of the sample were immediately deposited onto freshly cleaved micas. The micas were allowed to stand for 5 min and were then rinsed three times with double-distilled water (ddH_2_O) (100 μl each time) and dried in a gentle stream of nitrogen gas. For cross-linking reactions, before sample deposition, glutaraldehyde was added to reach a final concentration of 0.5%. After further incubation for 30 min at room temperature, the reaction was terminated by the addition of 50 mM Tris-Cl (pH 7.5). The sample was then dialyzed against 10 mM HEPES-KOH (pH 7.6)–100 mM KCl–2.5 mM MgCl_2_. Scanning in air was performed in ScanAsyst mode using a NanoScope V multimode AFM instrument (Digital Instruments, Santa Barbara, CA) under ambient conditions. Supersharp SacnAsyst silicon nitride tips (Bruker, USA) were used at a resonance frequency of about 70 kHz with a scanning rate of ∼1 Hz. The lengths of the naked DNA and the protein-bound DNA were measured manually using ImageJ Ver1.33u software (Wayne Rasband, National Institutes of Health, USA). Heights and widths of the condensed DNA structures of the Cren7-DNA complexes were determined by using the software provided with the NanoScope instrument. Typically, data sets of 30 molecules or complexes were obtained for each sample.

### Data accessibility.

All of the movies have been deposited in the China National Microbiology Data Center (accession number NMDCX0000001).
