# Supplementary figures and images for: Archaeal Chromatin Proteins Cren7 and Sul7d Compact DNA by Bending and Bridging
Source: mBio. 2020 Jun 9;11(3):e00804-20. doi: 10.1128/mBio.00804-20 (PMC7373190; doi:10.1128/mBio.00804-20)

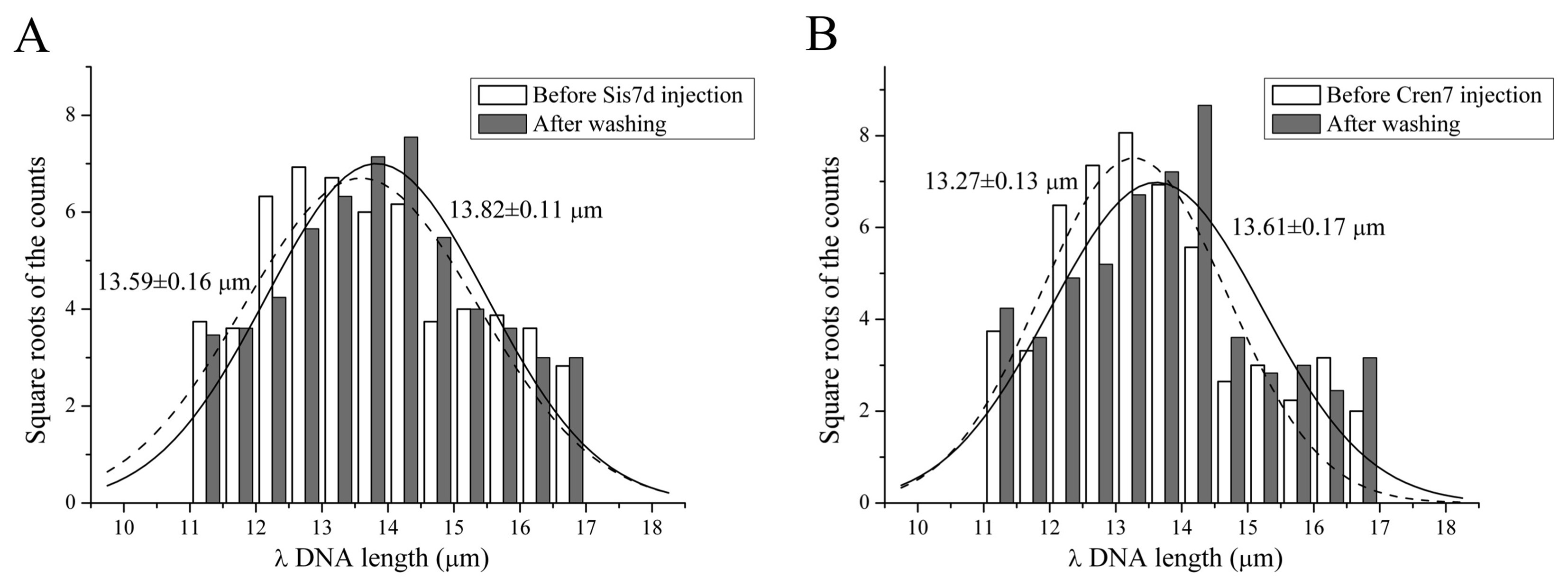

Supplement: FIG S1 [file mBio.00804-20-sf001.jpg]

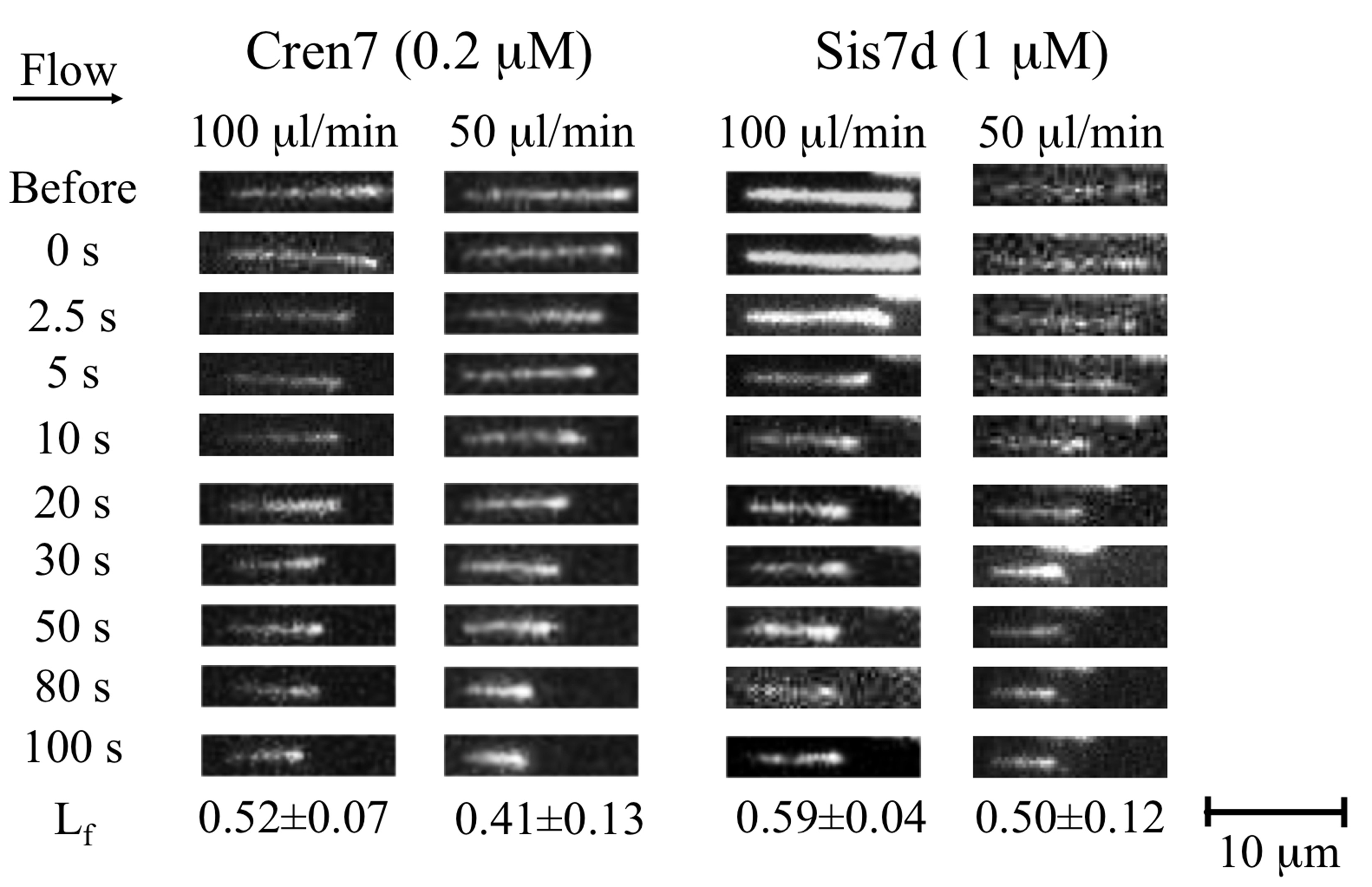

Supplement: FIG S2 [file mBio.00804-20-sf002.jpg]

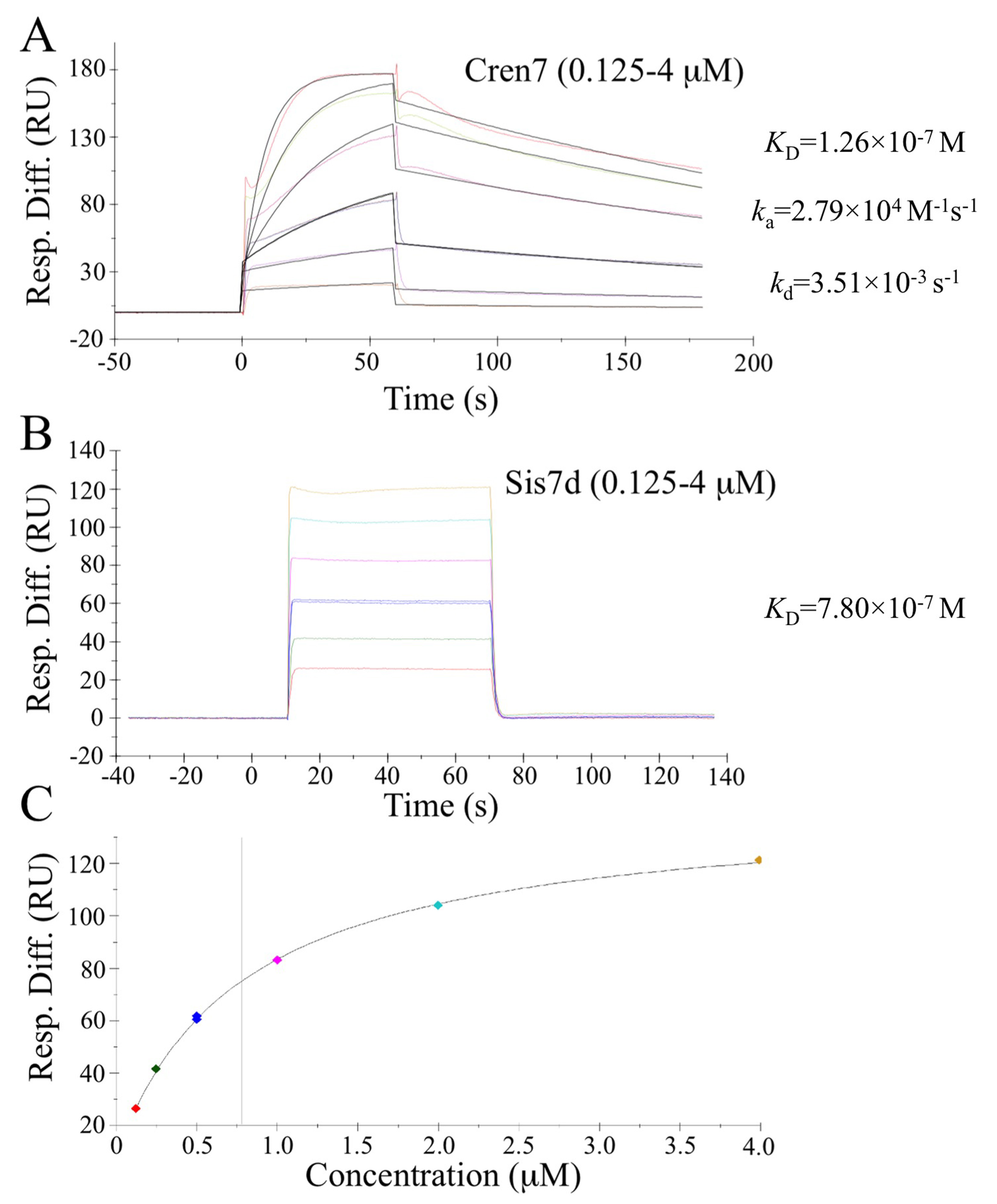

Supplement: FIG S3 [file mBio.00804-20-sf003.jpg]

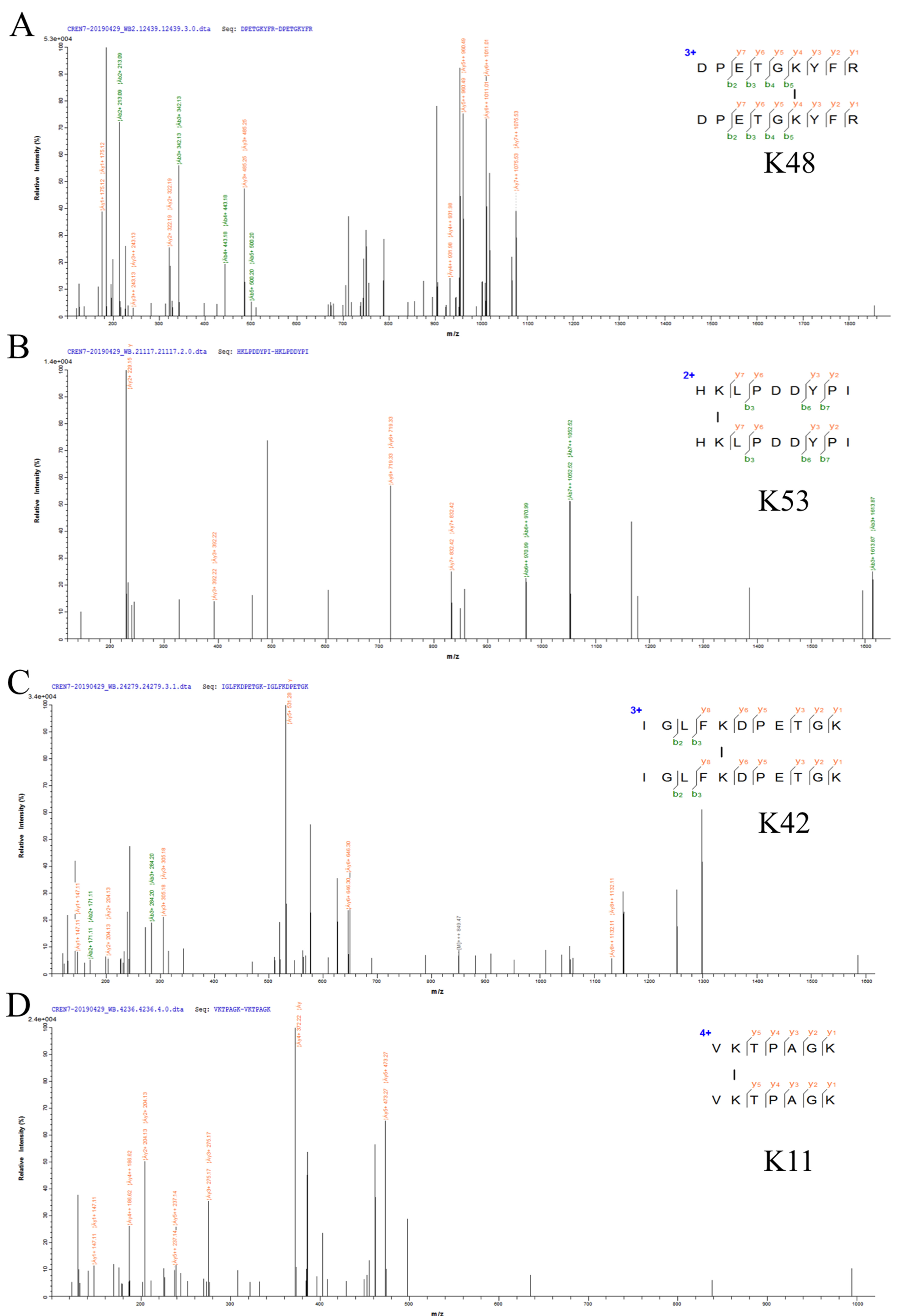

Supplement: FIG S4 [file mBio.00804-20-sf004.jpg]

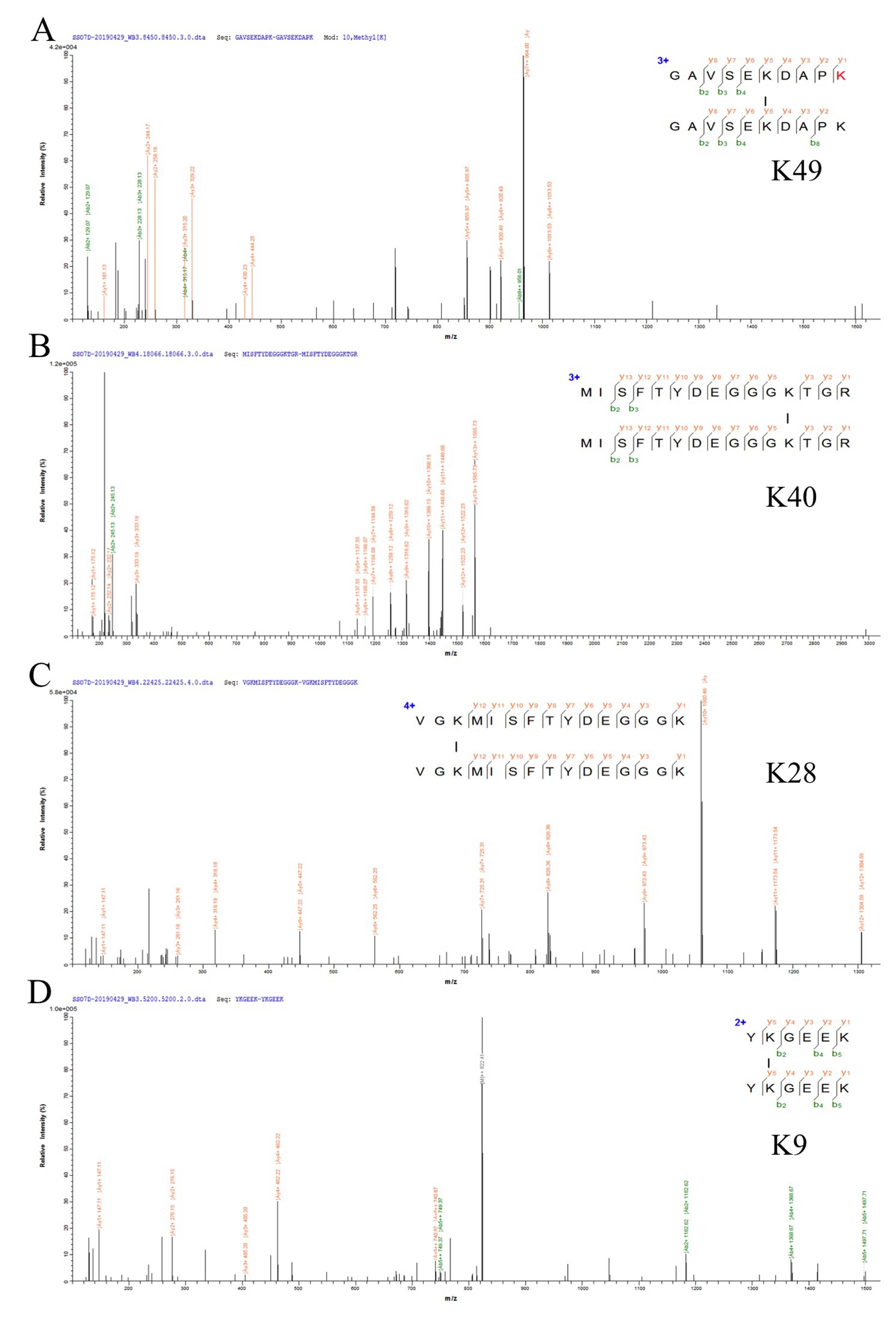

Supplement: FIG S5 [file mBio.00804-20-sf005.jpg]

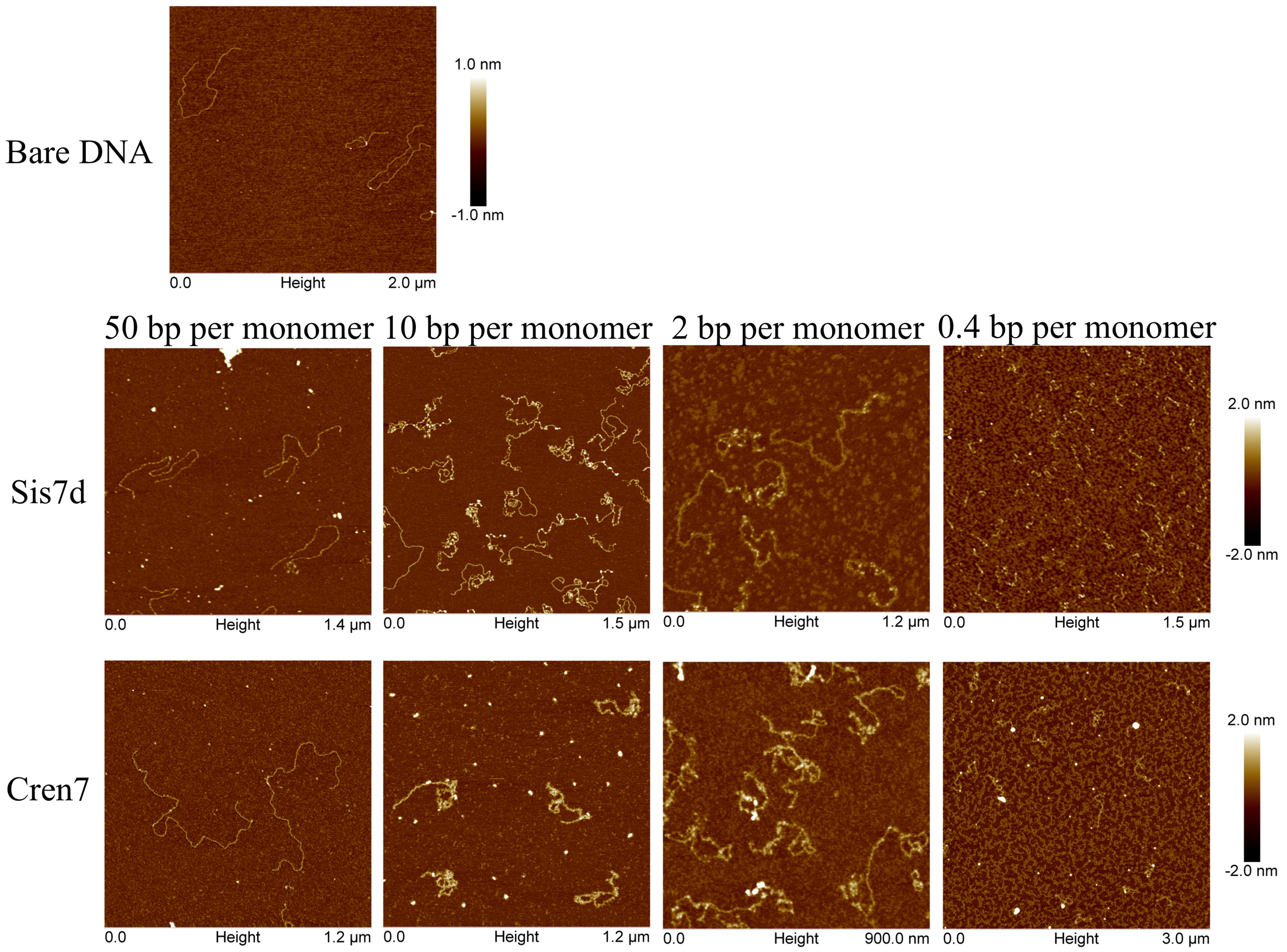

Supplement: FIG S6 [file mBio.00804-20-sf006.jpg]

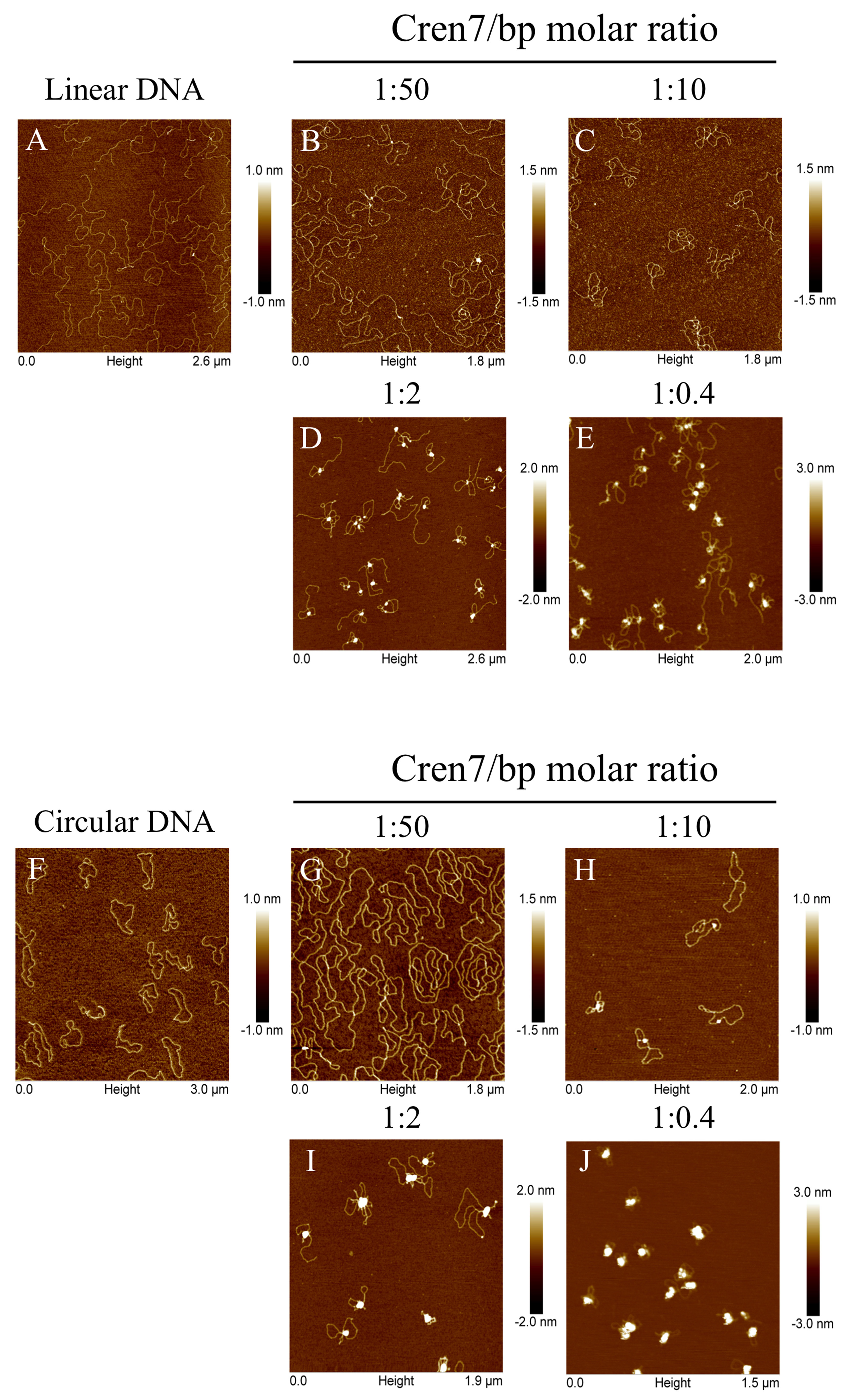

Supplement: FIG S7 [file mBio.00804-20-sf007.jpg]
